# Organoids for Metabolic Disease Modeling

**DOI:** 10.1002/jimd.70164

**Published:** 2026-03-10

**Authors:** Arif Ibrahim Ardisasmita, Edward Eelco Salomon Nieuwenhuis, Sabine Annemijn Fuchs

**Affiliations:** ^1^ Department of Metabolic Diseases Wilhelmina Children's Hospital, University Medical Center Utrecht Utrecht the Netherlands; ^2^ Regenerative Medicine Center Utrecht Utrecht the Netherlands; ^3^ Princess Máxima Center for Pediatric Oncology Utrecht the Netherlands; ^4^ United for Metabolic Diseases (UMD) the Netherlands

## Abstract

Inherited metabolic diseases (IMDs) are a diverse group of rare genetic disorders that disrupt metabolic pathways, leading to severe clinical manifestations. Disease models ranging from complex animal models to simple in vitro systems have provided insights into IMDs, but each has limitations. Organoids, three‐dimensional in vitro models, bridge this gap by replicating key metabolic functions that are absent in most simple 2D cell models. While organoids do not fully mimic organ complexity, they effectively model disease‐specific metabolic defects, as seen in methylmalonic acidemia, Wilson's disease, and cystic fibrosis. Recognizing that function is more critical than organ resemblance, we propose focusing on the specific function of interest rather than selecting a model solely based on its derivation from the most affected organ. Focusing on specific biological processes enables precise, disease‐relevant studies that drive novel therapeutic strategies and personalized medicine.

## 1 Introduction

### 1.1 Inherited Metabolic Diseases

Inherited metabolic diseases (IMDs) represent a large group of individually rare diseases that are characterized by alterations in metabolic processes in the body caused by genetic defects. Currently, more than 1450 diseases have been identified and this number is continuously growing, thereby also illustrating the large number of metabolic pathways that can be disrupted [1]. IMDs are highly heterogeneous in their presentation, as they can affect any organ in the body and manifest a diverse array of clinical symptoms at any age. Despite this high heterogeneity, the mechanism of disease is the same: genetic mutations leading to abnormal activity of the encoded protein, which leads to the disturbance of a metabolic pathway.

### 1.2 Disease Models for IMDs

To gain more insight in the effects of mutations on these metabolic pathways, and how resulting abnormalities may be targeted to develop therapies for IMDs, researchers use a variety of disease models. These disease models range from animal models to in vitro models with low levels of complexity. Disease models with high complexity, such as a whole organism (e.g., (humanized) animal models) or several organs/tissues (e.g., microphysiological systems or organ‐on‐a‐chip), allow researchers to examine disease phenotypes in a broader (human) context [2, 3]. Disease models with low complexity, such as 2D culture systems of patient‐derived cells or organ specific cancer cells, allow for more controlled experimental conditions and are ideal for uncovering detailed mechanistic insights.

### 1.3 The Importance of Functional Recapitulation

**FIGURE 1:**
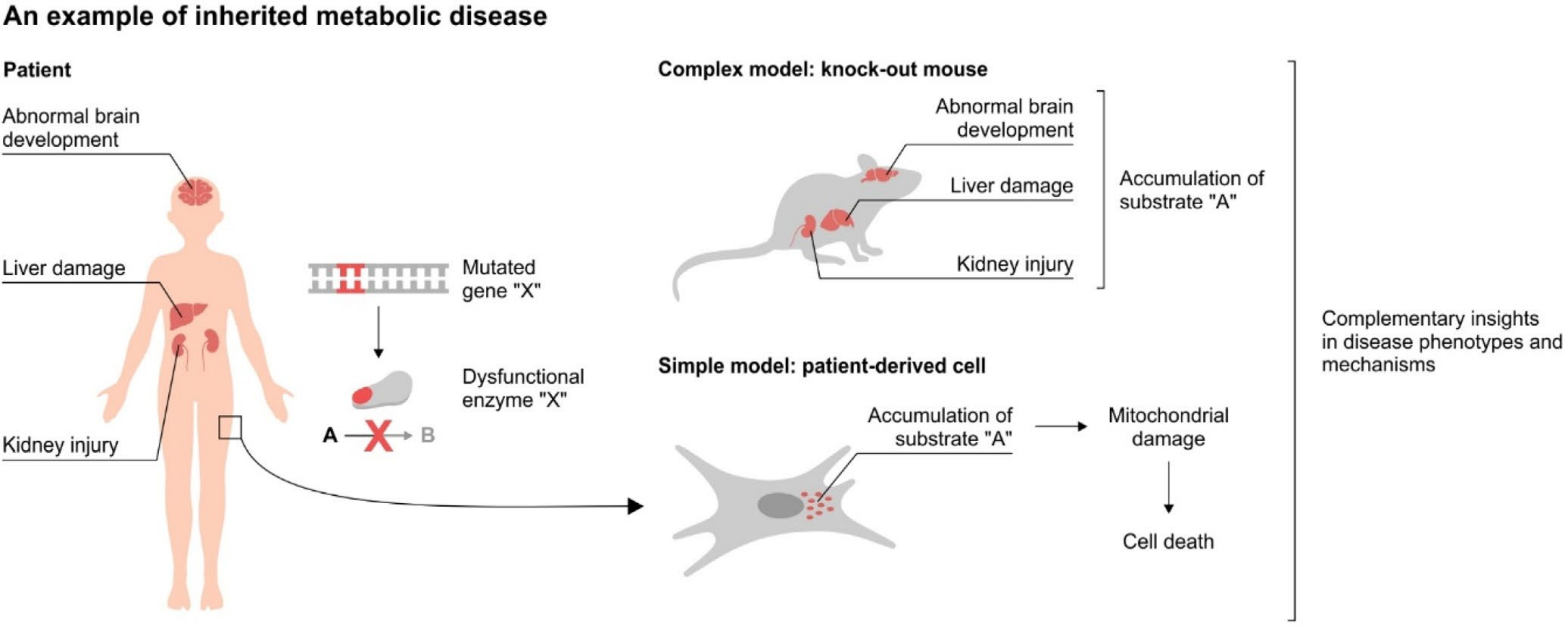
Complex and simple models can provide complementary insights on disease mechanism.

Both complex and simple disease models play important roles in elucidating disease mechanisms and discovering treatments for IMDs (Figure 1). As an example, galactosemia, an IMD caused by deficiencies of galactose metabolizing enzymes, has been studied extensively using both animal (complex) and cellular (simple) models (reviewed in [4]). Newborns with galactosemia are unable to metabolize galactose and may develop jaundice, cataracts, liver damage, kidney injury, and neurologic symptoms [5]. Using a galactose‐1 phosphate uridyltransferase‐deficient mouse model, accumulation of galactose metabolites was found in organs typically affected by the diseases such as the liver, kidney, and brain, leading to the hypothesized disease mechanism of metabolite accumulation [6]. Interestingly, this accumulation of galactose metabolites was also observed in patient‐derived cell lines such as fibroblasts [7], while skin symptoms are not characteristics of the disease. This led to the use of patient‐derived fibroblasts to shed light on other pathophysiological mechanisms including aberrant glycosylation and endoplasmic reticulum stress [8, 9].

The previous example shows how complex and simple models are complementary to each other. More importantly, this also illustrates how fibroblasts, a cell type that is not typically affected by galactosemia, can still be used as a valuable model provided that it accurately recapitulates the disrupted metabolic pathway. This underscores that the utility of a disease model hinges not on tissue origin but on its ability to reproduce the specific metabolic function of interest.

### 1.4 Organoids as the Ultimate Solution?

In the past decade, the use of organoids as disease models has gained momentum [10, 11, 12]. In terms of complexity, organoids are simpler than animal models but more complex than 2D cell lines. As the name implies, organoids are in vitro models that mimic organ structure and functions. In this sense, organoids provide a promising platform to study IMDs as a semi‐complex organ model that recapitulates the disease phenotype at an organ level and cell‐specific functions at the organ‐ and patient‐specific cellular level. Nevertheless, to what extent do organoids meet these expectations? Are organoids indeed “mini organs” that display full organ‐level complexity and functionality? Here, we will review current applications of organoids for disease modeling to determine their place in IMD research.

An example of an inherited metabolic disease (IMD) that causes abnormalities in patient brain, liver, and kidney. The disease is caused by a mutation in a gene leading to the expression of a dysfunctional enzyme, which is normally responsible for the metabolism of “A” to “B”. Using a knock‐out mouse model, accumulation of substrate “A” is found in the affected organs. However, how the accumulation leads to organ abnormalities remains unknown. Patient‐derived cells from an easily available, yet unaffected organ can be used to further study the disease if the cells recapitulate the relevant metabolic pathways. They can then be used to, for example demonstrate that accumulation of substrate “A” in the cells results in mitochondrial damage and ultimately cell death. The use of this simple cellular model allows investigation of cell‐specific response without being affected by processes from other and/or different cells. For example, accumulation of substrate “A” in the brain can be caused by a disrupted pathway in the brain cells or secondarily through overproduction of substrate “A” in liver cells. Hence, findings from both complex and simple models provide complementary insights in the disease mechanism.

## 2 Organoids

### 2.1 What Are Organoids?

Organoids are a three‐dimensional in vitro culture system established from self‐organizing stem cells, progenitor, or differentiated cells which mimic the architecture and functions of in vivo organs. They can be established from primary tissue or pluripotent stem cells (PSCs). Similar to in vitro models in general, organoids can present multiple degrees of complexity. Organoids can be made up of: (1) a single germ layer that comprises an organ (epithelial organoids); (2) multiple germ layers that constitute an organ (multi‐tissue organoids); or (3) germ layer(s) from multiple organs that self‐organize and interconnect (multi‐organ organoids) [13].

### 2.2 Do Organoids Recapitulate the Full Functionality and Structure of an Organ?

Being the simplest organoid system, epithelial organoids lack the cellular complexity of an organ. Epithelial organoids such as cholangiocyte organoids [17, 33] and hepatocyte organoids [34, 35] only recapitulate a subset of cells that make up the liver. Therefore, they do not reproduce the full functionality or structural complexity of the liver. Conversely, small intestinal organoids are capable of emulating numerous cell types and the architecture that makes up the epithelial lining of the small intestine [36]. Nevertheless, the epithelial intestinal organoid system still lacks the tubular structure, immune cells, muscle tissue, and vasculature to fully resemble the in vivo organ. Multi‐tissue organoids have been developed to address this limitation [37, 38], but they still do not demonstrate the full spectrum of organ cell/tissue diversity and functionality. Other approaches to enhance the organ‐specific cellular complexity of organoid systems include co‐culturing organoids with relevant supporting cell types [39, 40] or integrating organoid models with organ‐on‐chip technology [41, 42]. These strategies broaden physiological relevance by incorporating additional cellular interactions and microenvironmental cues, although they also introduce challenges such as balancing culture conditions for multiple cell types, maintaining control over differentiation, and interpreting more complex outcomes.

### 2.3 Do Organoid Cells Fully Replicate the Characteristics and Functions of Their in Vivo Counterparts?

Although currently, organoids do not completely recapitulate the functions and structure of the organ of interests, the cells that make up the organoids bear a certain degree of resemblance to their in vivo counterparts. The similarity between the organoid cells and the in vivo cells varies depending on the organoid type and the protocol used to generate the organoids. To illustrate, we previously performed a comprehensive comparison of hepatocyte in vitro models [43], including adult tissue derived intrahepatic cholangiocyte organoids (ICOs) [17], PSCs derived ICOs [44], and FHOs [35]. This thorough transcriptomic analysis shows that none of the in vitro models fully recapitulates the human primary hepatocyte phenotype. Instead, each model expresses a varying subset of specific hepatocyte functions. This analysis also revealed that ICOs, after being differentiated towards hepatocytes, are still more similar to cholangiocytes than hepatocytes. However, they are capable of recapitulating some hepatocyte functions such as various CYP activities and albumin secretion.

### 2.4 Are Organoids Useful to Study IMDs?

Despite not fully resembling the organ of origin, organoids are able to replicate specific organ functions. These functions may include specific metabolic or transport functions which are important for IMD modeling. An organoid system that faithfully represents the specific metabolic pathway of interest for an IMD can thus provide valuable insights. Moreover, organoids established from patient‐derived material enable the study of IMDs in a personalized manner.

The potential of organoids to model primary disease aspects of inherited diseases has been demonstrated (Table 1). ICOs grown from liver tissue from MMA patients had diminished enzymatic activity of methylmalonyl‐CoA mutase, resulting in the accumulation of propionyl carnitine [14, 18] and methylmalonic acid [18]. ICOs derived from Wilson's disease patients showed increased sensitivity to copper‐induced toxicity upon copper exposure, reflecting reduced copper export capacity due to the mutated *ATP7B* gene [14]. Similarly, kidney tubuloids from cystinotic patients displayed the disease phenotype with cystine accumulation, which responded to treatments typically used in patients [20].

**TABLE 1:** Examples of inherited diseases modeled using organoids.

Disease	Affected genes	Affected functions	Organoid models	References
Wilson's disease	*ATP7B*	Copper transport	ICOs	[14]
Cystic fibrosis	*CFTR*	Anion transport	Intestinal organoids	[15]
			Rectal organoids	[16]
Alpha‐1 antitrypsin (AAT) deficiency	*SERPINA1*	AAT secretion	ICOs	[17]
Methylmalonic acidemia	*MMUT*	Branched‐chain amino acid metabolism	ICOs	[14, 18]
Acid sphingomyelinase deficiency	*SMPD1*	Sphingomyelin accumulation	ICOs	[19]
Cystinosis	*CTNS*	Cystine transport	Kidney tubuloids	[20]
Fabry disease	*GLA*	Alpha‐galactosidase A activity	Kidney organoids	[21, 22]
Stargardt disease type 1	*ABCA4*	ABCA4 expression and localization	Retinal organoids	[23]
Phosphomannomutase 2 congenital disorders of glycosylation (PMM2‐CDG)	*PMM2*	Glycosylation	Cortical organoids	[24]
Vacuolar protein sorting 45 homolog (VPS45) deficiency	*VPS45*	Myelofibrosis	Bone marrow organoids	[25]
Ornithine transcarbamylase deficiency	*OTC*	Urea cycle	Liver organoids	[26]
			Hepatocyte organoids	[27]
Crigler–Najjar syndrome	*UGT1A1*	Bilirubin metabolism	Liver organoids	[28]
Niemann‐Pick disease type C	*NPC1/NPC2*	Lysosomal cholesterol accumulation	Brain organoids	[29]
CLN3 deficiency	*CLN3*	Phospholipid metabolism	Cerebral organoids	[30]
Glycogen storage disease type Ia (GSD‐Ia)	*G6PC*	Gluconeogenesis	Hepatocyte organoids	[27]
Leigh syndrome	*SURF1*	Oxidative phosphorylation	Cerebral organoids	[31]
Krabbe disease	*GALC*	Galactosylceramide breakdown	Myelinating organoids	[32]

The unique ability of organoids to capture organ‐specific functions makes them particularly valuable for investigating organ‐specific symptoms of IMDs. For example, in Krabbe disease, fibroblasts and lymphocytes can be used to assess the activity of the affected enzyme [45], but they cannot reproduce tissue‐specific phenotypes such as demyelination. In contrast, myelinating organoids provide an ideal model to study this key pathological feature [32]. Organoids also offer the potential to investigate organ‐specific differences in IMD symptoms and pathogenesis. For example, MMA causes mitochondrial dysfunction in both proximal tubules and neurons. However, the downstream consequences differ by organ: in the kidney, mitochondrial dysfunction primarily drives damage through increased oxidative stress, whereas in neurons it manifests as a neuronal exhaustion‐like phenotype [46, 47].

The 3D structure of organoids can offer a major advantage, which was exploited for cystic fibrosis (CF) modeling in intestinal organoids [15]. Intestinal organoids are cultured with forskolin, an inducer of cyclic AMP which activates the cystic fibrosis transmembrane conductance regulator (CFTR) protein. In healthy organoids, activation of CFTR leads to transport of fluid into the lumen of organoids, causing them to swell. In contrast, organoids from CF patients show reduced forskolin‐induced swelling (FIS) and the magnitude of the swelling impairment correlates with the disease severity associated with the different genetic mutations. This swelling can be reliably quantified in patient‐derived organoids and this has led to the Forskolin‐induced swell (FIS) assay to become an invaluable tool to study CF, to develop and test novel therapies and to guide personalized treatment [16, 48].

We illustrate the shift in focus from organ resemblance to functional recapitulation using liver‐derived organoids, though this concept is broadly applicable to organoids derived from any tissue. Liver‐derived organoids are seen as in vitro models that resemble the liver. However, they do not fully mimic the liver due to lack of cellular complexity and incomplete organ function recapitulation. The cells that make up the organoids also remain deficient in completely mimicking there in vivo counterparts. By shifting the perspective to functional recapitulation, the focus is on the functions the model can perform, regardless of where those functions occur in the body. For example, liver‐derived organoids can model copper transport and metabolism (a key liver function), branched‐chain amino acid metabolism (performed by both liver and brain), and CFTR‐mediated transport (typically associated with the lung). By prioritizing functional capability over organ resemblance, the perspective of functional recapitulation offers a more targeted and effective framework for studying IMDs.

Additionally, organoid models are highly valuable for studying gene correction strategies in IMDs when they comprise the patient‐specific genome (including mutations) and the resulting functional impairments. For instance, Schene et al. [49] achieved genetic and functional correction of diacylglycerol acyltransferase 1 (DGAT1) deficiency and Wilson's disease in patient‐derived organoids using prime editing. These models exhibited disease‐relevant functional disruptions, which resolved following gene correction. Similarly, Bulcaen et al. [50] applied prime editing to cystic fibrosis patient‐derived rectal organoids and nasal epithelial cells, demonstrating genetic and functional restoration in both systems. Organoids have also been widely used to demonstrate genetic and functional correction of base editing's [51, 52].

### 2.5 From Full Organ Resemblance to Functional Recapitulation

#### 2.5.1 Shifting the Focus From Complete Organ Identity to Functional Fidelity

**FIGURE 2:**
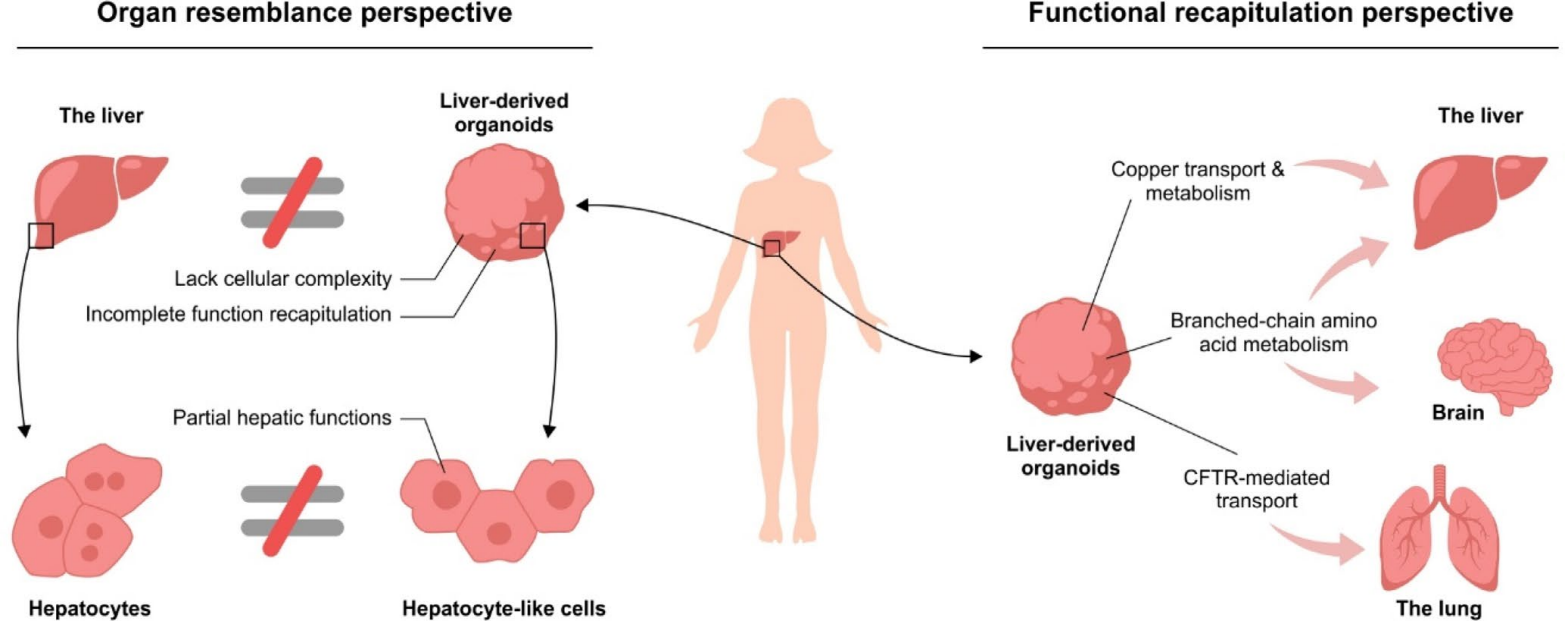
The perspective shift from a focus on organ resemblance to functional recapitulation.

Organoids often fall short in fully replicating the complexity of their organ of origin but can effectively replicate specific organ functions, making them especially valuable for studying IMDs. The functional perspective focuses on the biological functions that an organoid model recapitulates rather than the complete structural or cellular resemblance to the native organ (Figure 2). This perspective prioritizes functional activity as the primary criterion for evaluating and applying organoid models in IMD research (Table 2).

**TABLE 2:** Comparison between organ resemblance vs. functional recapitulation perspective.

Aspect	Organ resemblance perspective	Functional recapitulation perspective
Evaluation Basis	Organ resemblance (morphology, transcriptome, markers).	Functional pathway activity, independent of organ resemblance.
Model Selection	Organ‐matched models (e.g., liver organoids for liver disease).	Any model that robustly capture the function of interest.
Complexity Requirement	High structural and cellular complexity assumed necessary.	Minimal complexity sufficient if function can be measured.
Limitations	Assumes faithful organ/cell recapitulation; May overlook simpler but functional models.	May miss organ‐specific context or unknown modifying pathways
Core Question	Which organ does this model resemble?	Which function does this model recapitulate?
Typical Use Case	Studying organ‐specific pathophysiology.	Studying pathway activity, functional restoration, or prediction.
Application Example	Myelinating organoids to study Krabbe disease myelination (organ specific) [32].	Rectal organoids to measure CFTR function and predict CF therapy response [53].

While IMDs may present with organ‐specific symptoms, this organ specificity is a reflection of the underlying metabolic functions disrupted in those organs. Therefore, the organ‐specific context in IMDs can also be viewed as a collection of relevant functional processes rather than strict anatomical localization.

This raises an important question: how complex must the replication of these organ‐specific functional processes be to effectively model IMDs? Is it necessary to recapitulate the full functional complexity of the organ, or is it sufficient to reproduce a specific subset of critical metabolic functions? We believe that functional models should not be more complex than necessary to accurately investigate the function of interest. Inherently, this depends on the specific research question, the underlying disease mechanism, and the functional relevance of the targeted and related pathways to the clinical phenotype.

### 2.6 Disease Modeling Through the Functional Recapitulation Perspective

By applying the functional recapitulation perspective, organoid models are primarily selected based on their ability to recapitulate the critical function affected by the disease, rather than their anatomical origin (Figure 2). For example, in CF, the key function of interest is CFTR‐mediated ion transport. Although CF primarily affects the lungs, pancreas, and liver, organoids derived from the easily accessible human rectum are frequently used to model the disease [15, 16]. These rectum‐derived organoids faithfully recapitulate CFTR function within a 3D culture system, making them suitable for the FIS assay, a widely adopted tool in CF research and personalized therapy development. Importantly, results from the FIS assay using patient‐derived rectal organoids have been shown to correlate with drug efficacy and clinical disease features and severity in patients [16, 53, 54, 55], underscoring the validity and value of function‐based model selection.

The functional recapitulation perspective is particularly valuable for assessing functional restoration after gene editing in IMDs. Most genetic mutations underlying IMDs result in defective protein function, often leading to the accumulation of toxic metabolites. Evaluating the success of gene editing therefore primarily requires measuring the restoration of the affected protein's activity. As long as the relevant protein is expressed in the organoid model, functional assays can be performed even without complete recapitulation of the diseased organ's full complexity [49].

**FIGURE 3:**
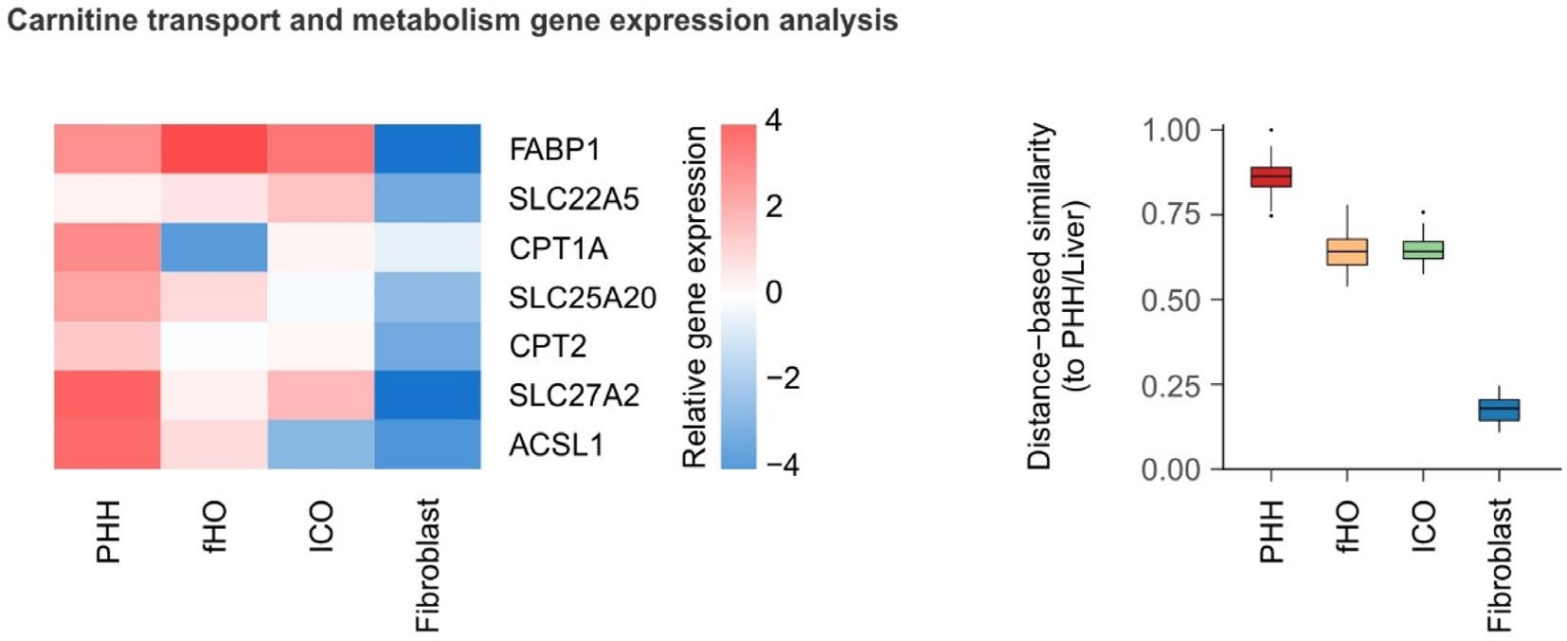
Despite only partial liver resemblance, cholangiocyte organoids recapitulate carnitine transport and metabolism to a similar extent as primary human hepatocytes and hepatocyte‐derived organoids based on gene expression analysis.

Selection of an organoid model to study an IMD should be guided by the extent to which it recapitulates the function of interest. As an example, when studying carnitine palmitoyltransferase 2 (CPT2) deficiency, the most important aspect is recapitulation of carnitine transport and metabolism. Although the main affected cell types are hepatocytes, cardiomyocytes, and skeletal muscle cells [56], ICOs recapitulate carnitine transport and metabolism at a gene expression level comparable to that of both primary human hepatocytes (PHHs) and FHOs (Figure 3). This indicates that ICOs can serve as a relevant model for studying CPT2 deficiency, despite their closer resemblance to cholangiocytes than to hepatocytes. This example also underscores the importance of evaluating not only the affected gene but also the broader metabolic pathway in which the gene functions. In the case of CPT2, its function cannot be effectively modeled unless other components of the pathway, such as the transporters OCTN2 (SLC22A5) and CACT (SLC25A20), as well as upstream enzymes like CPT1 and CPT1A, are also sufficiently expressed and functional [57]. Depending on the importance of specific associated genes, an informed choice between using FHOs or ICOs can be made to best support the study of the disease mechanism (Figure 3). However, if the aim of the study is to investigate the mechanism by which CPT2 deficiency may lead to arrhythmia [58], the model must be able to recapitulate functions relevant to cardiac electrophysiology. In such a case, ICOs or FHOs, despite their ability to model carnitine metabolism, would not be appropriate, as they lack the cardiomyocyte‐specific functions required to study arrhythmic phenotypes. This further reinforces the functional recapitulation perspective: model selection should be driven by the specific function under investigation.

### 2.7 Limitations Associated With the Functional Recapitulation Perspective

The functional recapitulation perspective enables precise investigation of specific biological processes by focusing on discrete functional units relevant to the disease. However, the effectiveness of this simplified approach depends on a comprehensive understanding of the metabolic dysfunction and its recapitulation within the in vitro model. If key aspects of the disease mechanism are not fully understood, particularly interactions with unknown or poorly characterized pathways, there is a risk that these components may not be recapitulated, potentially leading to misinterpretation of the disease phenotype and underlying mechanisms.

To mitigate the risk of missing relevant biological processes due to reduced model complexity or the absence of context‐dependent interactions, thorough validation of an in vitro model's predictive capacity is essential. Ideally, this validation is achieved by benchmarking functional readouts against relevant clinical data. The extensive validation of the FIS assay for predicting clinical outcomes in CF patients provides a strong example of how functional readouts can be rigorously assessed for translational relevance [16, 53, 54, 55].

Additionally, this limitation can be addressed by employing models that offer a higher degree of organ‐level recapitulation or cellular complexity. Organ‐specific organoid models, co‐culture models, or organ‐on‐a‐chip technologies may better capture the full spectrum of physiological interactions, including those involving unknown or secondary pathways. Such complex models increase the likelihood of retaining emergent properties and unanticipated interactions that are critical for faithful modeling of disease phenotypes. While they may be less targeted, these comprehensive systems serve as valuable complements, particularly in cases where the disease mechanism is incompletely understood or involves multi‐lineage crosstalk. Nevertheless, although more complex, these culture systems are still far from the complex human body and validation is required for extrapolation to in vivo settings.

RNA sequencing comparison using HLCompR analysis (https://utrecht‐university.shinyapps.io/HLCompR/) of primary human hepatocyte (PHH), fetal hepatocyte organoid (FHO), intrahepatic cholangiocyte organoid (ICO), and fibroblast samples [43]. (Left) Heatmap showing the expression of genes related to carnitine transport and metabolism. (Right) Similarity level for each sample to PHH/Liver as analyzed using HLCompR with the carnitine transport and metabolism gene set. Further selection of the model can be done based on the importance of *CPT1A* vs. *ACSL1* expression, resulting in a preference for either ICOs or FHOs, respectively.

## 3 Conclusions and Future Perspectives

IMDs represent a very diverse group of diseases in which a large number of metabolic pathways can be disrupted. While referred to as monogenic diseases, the monogenic defect generally results in a complex metabolic rewiring which can contribute to the disease manifestations. To study IMDs, robust disease models are required that can recapitulate key aspects of the disrupted and associated metabolic pathways. Simple models, such as patient‐derived fibroblasts, have provided foundational insights, but their utility is limited by their inability to replicate all disease‐relevant functions. Complex models, like animal models, offer the advantage but also the complexity of involving a complete organism, which, in addition, is not human. Organoids represent a versatile platform that balances complexity and functionality. These three‐dimensional cultures have demonstrated their potential to model disease‐specific metabolic functions, such as those seen in methylmalonic acidemia, Wilson's disease, and cystic fibrosis. The ability of organoids to provide personalized insights, as seen in cystic fibrosis FIS assays, exemplifies their transformative role in translational research. Additionally, organoids can be further refined to model more complex disease mechanisms by co‐culturing them with other cell or organoid types.

However, the field should consider prioritizing functional fidelity over organ resemblance, especially when studying well‐defined functions where broader pathway interactions are known or not of interest, thereby emphasizing the functional recapitulation perspective. By focusing on specific biological processes, this perspective redefines the role of organoids in IMD research. These specialized systems enable precise, disease‐relevant investigations, paving the way for personalized medicine and the development of pre‐clinical testing of novel therapeutic approaches. While challenges remain in fully replicating the complexity of human organs and cell types, the continuous evolution of organoid technology ensures a promising future for IMD modeling and treatment development. Development of technologies that support the characterization and selection of organoid models for specific functions will propel the implementation of the optimal organoids for individual research studies, as we have demonstrated using HLCompR. We believe that shifting the focus from organ modeling to simpler function‐based modeling offers the most effective approach to studying IMDs.

## Author Contributions

Conceptualization: A.I.A., E.E.S.N., and S.A.F.; writing – original draft: A.I.A.; writing – review and editing: A.I.A., E.E.S.N., and S.A.F.; visualization: A.I.A.; supervision: E.E.S.N. and S.A.F.; and funding acquisition: E.E.S.N. and S.A.F.

## Funding

This work was supported by ZonMw, (2021/15188/ZONMW), Stichting Metakids (2017‐072), European Research Council (10104161).

## Conflicts of Interest

The authors declare no conflicts of interest.

## Data Availability

Data sharing not applicable to this article as no datasets were generated or analyzed during the current study.
